# Multimodality Evaluation of Spontaneous Coronary Artery Dissection in a Patient With Connective Tissue Disease: A Case Report

**DOI:** 10.1016/j.jscai.2023.101125

**Published:** 2023-08-25

**Authors:** Tayyab Shah, Nimish N. Shah, Lauren A. Baldassarre, Lisa Freed, Hamid Mojibian, Samit Shah

**Affiliations:** a Yale School of Medicine, New Haven, Connecticut; b VA Connecticut Healthcare System, West Haven, Connecticut

**Keywords:** cardiac magnetic resonance imaging, coronary function testing, myocardial infarction with no obstructive coronary artery disease, spontaneous coronary artery dissection, vasospasm

## History of presentation: how the patient was admitted and physical examination

A 62-year-old woman with hyperlipidemia, obstructive sleep apnea, undifferentiated connective tissue disease complicated by recurrent pericarditis, and Raynaud’s phenomenon who was taking chronic prednisone, hydroxychloroquine, and interleukin 1 inhibitors presented to the emergency department with 3 days of persistent substernal chest pain after a recent, 5-day interruption in interleukin 1 inhibitor therapy.

The patient reported the chest pain began while climbing stairs and persisted for 3 days. The pain was sharp, constant, radiating to the neck and shoulders, exacerbated by inspiration and chest wall palpation, and improved with leaning forward. These symptoms were reminiscent of her multiple prior episodes of pericarditis, which last occurred more than a year prior to admission. She also reported nausea, shortness of breath, and diaphoresis. Physical examination revealed normal vital signs, no evidence of volume overload, and normal heart sounds with no friction rub.

## Differential diagnosis

Given the patient’s medical history and description of pain, there was high suspicion of pericarditis. However, with multiple risk factors for obstructive coronary artery disease (CAD) or a nonatherosclerotic coronary vasomotor disorder, acute coronary syndrome was also considered. Noncardiac causes of chest pain including musculoskeletal pain, pleuritis, and pulmonary embolism were considered less likely differential diagnoses.

## Investigations

**Figure 1:**
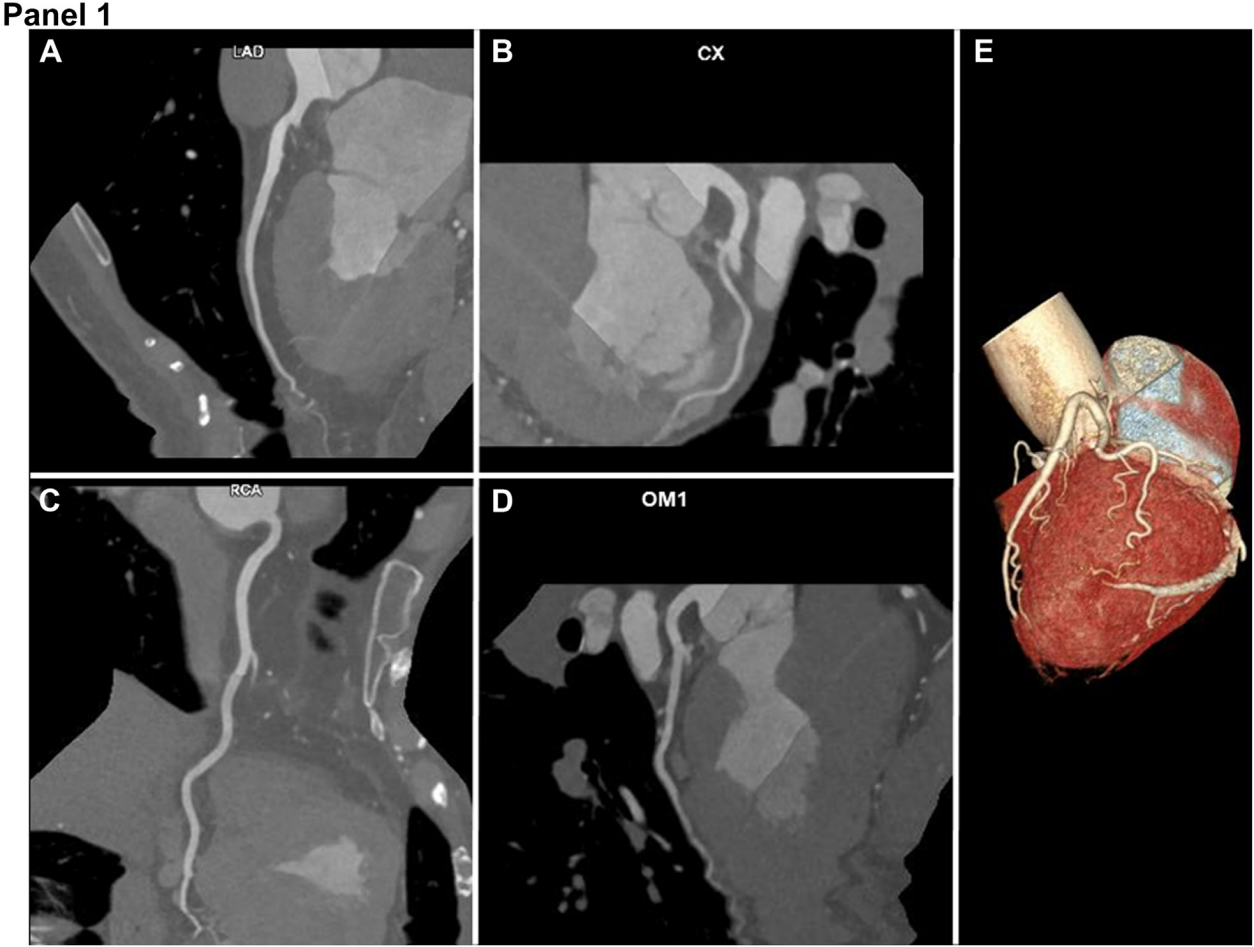

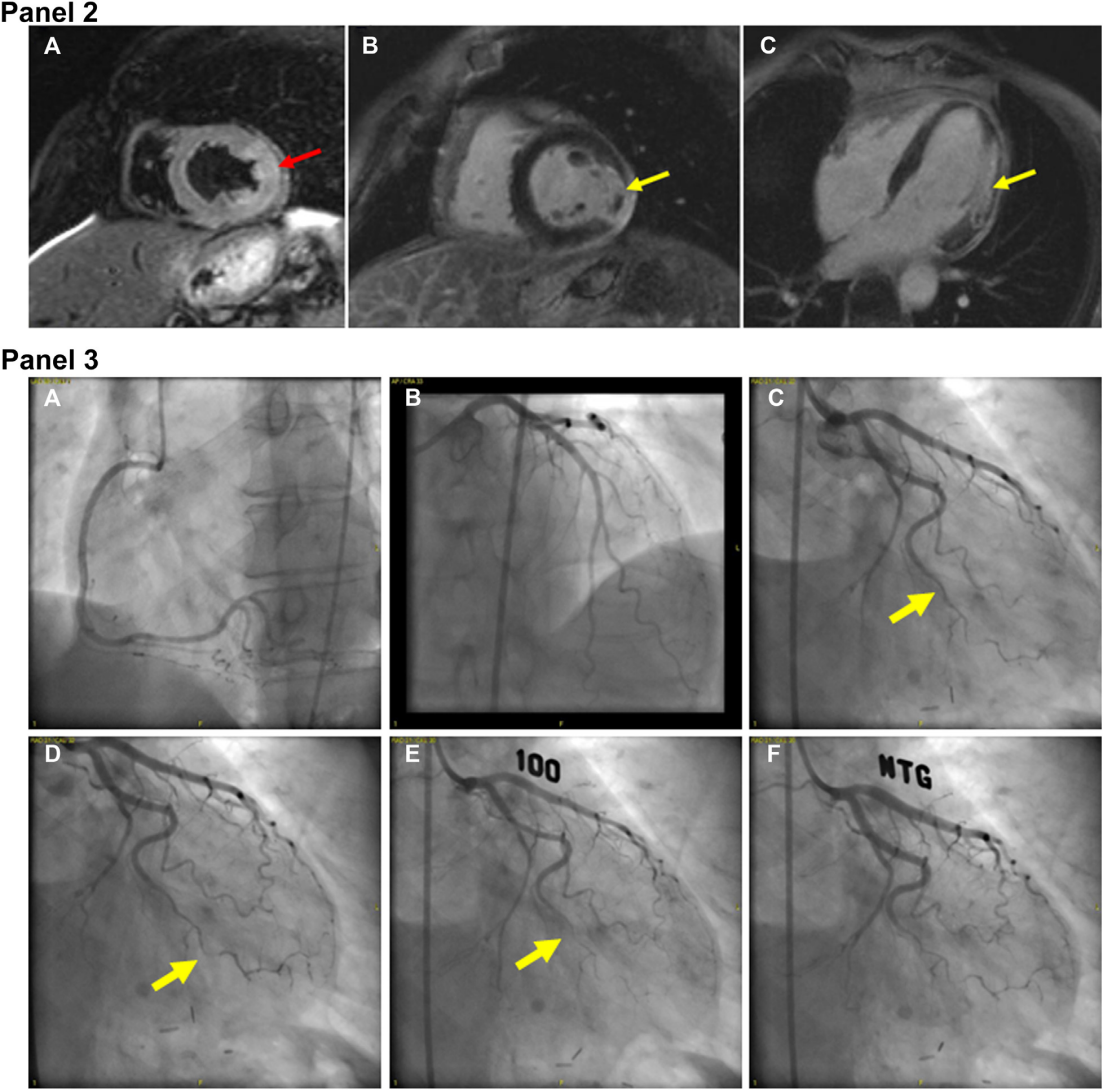
**Coronary computed tomography angiogram, cardiac magnetic resonance, and invasive coronary angiogram images of the patient.Panel 1.** Representative coronary computed tomography angiogram multiplanar reformatted images representing the left anterior descending (**A**), left circumflex (**B**), right (**C**), and obtuse marginal (**D**) coronary artery territories. The first obtuse marginal artery is also demonstrated on 3D volume-rendered reconstruction (**E**). **Panel 2.** Cardiac magnetic resonance imaging. T2-weighted signal acquisition demonstrating diffuse enhanced T2 signal in the mid lateral wall (**A**, red arrow), which also extends to the apical lateral segment (not shown). Short-axis (**B**) and 4-chamber (**C**), postcontrast acquisition after infusion of gadoterate meglumine demonstrating transmural late gadolinium enhancement in the mid lateral segments with central signal hypointensity (yellow arrow) and adjacent delayed pericardial enhancement. **Panel 3.** Invasive coronary angiogram, demonstrating no atherosclerotic disease in the right coronary (**A**), left anterior descending (**B**), or left circumflex (**C**) arteries, yet with evidence of dissection (**C**, arrow) and spasm (**D**, arrow) of the distal first obtuse marginal (OM1) artery. With 100 μg intracoronary acetylcholine, symptomatic vasospasm of the OM1 (arrow) occurred with 1-mm ST-segment depression (**E**) and was relieved with intracoronary nitroglycerin (**F**).

On presentation, high-sensitivity troponin T (hs-TnT) was elevated to 168 ng/L (normal <12 ng/L) and was 163 ng/L after 3 hours. An electrocardiogram showed normal sinus rhythm with new inferolateral T-wave inversions, which resolved and intermittently reoccurred during admission. Transthoracic echocardiography showed a left ventricular ejection fraction of 59% without regional wall motion abnormalities or pericardial effusion. Given initial concern for acute coronary syndrome, she underwent a coronary computed tomography angiogram (CCTA), which demonstrated normal coronary anatomy with no evidence of atherosclerotic CAD. A small change in lumen caliber was seen in the mid to distal obtuse marginal; however, no plaque or other etiology of lumen caliber change was visualized (Figure 1, Panel 1). Despite improving hs-TnT, the patient continued to have chest pain. Cardiac magnetic resonance imaging (CMR), including dynamic steady state free precession cine, T2-weighted, and late gadolinium enhancement imaging was performed on day 5 of hospitalization. CMR revealed mildly depressed left ventricular ejection fraction of 51%, akinesis of the mid to apical lateral segments, increased T2 signal intensity in the entire lateral wall, and transmural late gadolinium enhancement in the mid lateral segments with hypointense central signal, consistent with acute myocardial infarction (MI) with microvascular obstruction. There was also delayed pericardial enhancement adjacent to the MI territory suggesting peri-infarct pericarditis (Figure 1, Panel 2). Invasive coronary angiography was performed the following day, demonstrating no atherosclerotic lesions (Figure 1, Panels 3A-C); however, the distal segment of the first obtuse marginal was abruptly narrowed, suggesting spontaneous coronary artery dissection (SCAD) (Figure 1, Panel 3C, arrow). Coronary function testing was performed with acetylcholine provocation and coronary thermodilution. Injection of 100 μg of intracoronary acetylcholine provoked vasospasm of >90% of the distal first obtuse marginal (Figure 1, Panels 3D-E), which was accompanied by chest pain and 1-mm ST depressions in the lateral leads. The patient’s symptoms, electrocardiogram changes, and angiographic spasm were relieved by administration of 200 μg of intracoronary nitroglycerin (Figure 1, Panel 3F). Fractional flow reserve of the left anterior descending artery was normal, yet thermodilution revealed an abnormal index of microcirculatory resistance of 39, consistent with coronary microvascular dysfunction. The patient was thus diagnosed with acute lateral wall myocardial infarction with no obstructive coronary artery disease (MINOCA) secondary to SCAD in the setting of vasospasm and microvascular dysfunction with associated peri-infarct pericarditis.

## Management (medical/interventions)

Given that the CCTA was negative for obstructive CAD on admission, the patient was initially treated for presumed myopericarditis with ibuprofen, colchicine, and an increase in her chronic prednisone dose. Once SCAD and vasospasm were diagnosed, the patient was started on aspirin and a dihydropyridine calcium channel blocker, ibuprofen was stopped, and prednisone was rapidly tapered. Given the strong association between fibromuscular dysplasia and SCAD,^1^ computed tomography angiography of the abdomen and pelvis was also performed and demonstrated no abnormality in the visceral vessels.

## Discussion

MINOCA occurs due to a heterogenous group of syndromes that are often underdiagnosed and undertreated, including primary vascular pathologies, such as SCAD.^2^^,^^3^ In this patient, there was initially a high index of suspicion for acute MI related to atherosclerotic CAD. However, CCTA showed no significant atherosclerosis, and a transthoracic echocardiogram did not reveal wall motion abnormalities. Although CCTA is a promising technology for the diagnosis of SCAD, there are limited data on the test characteristics of CCTA, especially for distal vessel involvement. Recent case series have identified various features to assist in the diagnosis of SCAD using CCTA,^4^^,^^5^ but these reports also demonstrated patients who had normal CCTA but had SCAD eventually diagnosed on invasive angiography. This may be due to the limited resolution of CCTA to exclude dissection in smaller distal vessels, as in this case. Consequently, particularly in patients with risk factors for SCAD (eg, female sex, underlying connective tissue disorder, and prior SCAD) and persistent symptoms, invasive angiography should still be considered for definitive diagnosis.^2^

In the reported case, CMR was an invaluable tool to understand the pathophysiology of the patient’s chest pain and reveal an underlying ischemic process. In the evaluation of patients with suspected cardiac chest pain and elevated troponin but low suspicion of CAD, CMR can noninvasively distinguish ischemic vs nonischemic syndromes, as it did in our case. Prior studies have demonstrated the significant improvement in diagnosis by combining comprehensive coronary evaluation and CMR for patients with MINOCA,^6^ and recent analyses support early CMR utilization in the diagnostic algorithm for evaluation of MINOCA.^7^ In our case, the diagnostic coronary angiogram after CMR confirmed the diagnosis of SCAD, which explained the clinical and imaging evidence of MI. However, the patient’s intermittent inferolateral T-wave inversions, recurrent episodes of chest pain without rising hs-TnT, and improvement in her symptoms with nitroglycerin suggested that her symptoms were not fully explained by pericarditis or MI from SCAD. Because of this, coronary function testing was also pursued, which revealed vasospasm in the territory of the dissection and fully explained the clinical presentation. Many patients with SCAD have recurrent symptoms after the index dissection, and this comprehensive evaluation during her initial angiogram confirmed her underlying diagnosis and limited her exposure to subsequent procedures. Thus, in our case, a multimodality approach using CMR and invasive vasoreactivity and coronary physiology testing to efficiently differentiate additional causes of MINOCA helped identify the underlying diagnoses.

## Follow-up

After discharge, the patient continued to have intermittent mild chest pain requiring augmentation of her amlodipine dose and initiation of isosorbide mononitrate. She also completed cardiac rehabilitation and has been symptom free for >6 months without reoccurrence of SCAD. Her ejection fraction returned to baseline on follow-up outpatient echocardiogram.

## Conclusion

MINOCA is a challenging clinical entity for which multimodality imaging, including CMR and invasive angiography with vasoreactivity testing and coronary physiology testing, may aid diagnostic accuracy.
